# Supplementary material for: Are carbon emissions trading and green financial instruments synergistic? -Comprehensive quantitative research based on content analysis
Source: PLoS One. 2024 Mar 7;19(3):e0298601. doi: 10.1371/journal.pone.0298601 (PMC10919601; doi:10.1371/journal.pone.0298601)
Supplement: S1 Data — (ZIP) [file pone.0298601.s001.zip › Data/地级市Z_F工作报告.zip!/╣≤╓▌/░▓╦│╩╨/└┤╫╘╣1⁄2╓┌║┼í╛┬φ┐╦╩2╛▌═°í┐_2012.docx]

各位代表：

现在，我代表市人民政府向大会作工作报告，请予审议，并请市政协委员和其他列席会议的同志提出意见。

一、过去五年工作回顾

五年来，市人民政府在省委、省政府和市委的领导下，在市人大、市政协的监督、支持下，坚持以邓小平理论和“三个代表”重要思想为指导，全面贯彻落实科学发展观，紧紧抓住西部大开发和新阶段扶贫开发的机遇，团结和依靠全市各族人民，开拓创新、锐意进取，克服了全球性金融危机、百年不遇的冰冻雪凝灾害和特大干旱带来的不利影响，胜利完成了市二届人大一次会议确定的目标任务，经济持续健康发展，社会事业全面进步，人民生活水平明显提高，社会主义民主法制和精神文明建设取得新成绩。

——经济实力逐步增强。预计全市生产总值由2006年的129.12亿元提高到2011年的286亿元，年均增长12.6%；人均生产总值由5156元提高到12190元，年均增长14.1%。财政总收入、地方财政一般预算收入分别由2006年的18.38亿元、9.73亿元提高到2011年的60.8亿元、24.89亿元，年均分别增长27%、20.7%。全社会固定资产投资由2006年的67.82亿元提高到2011年的258亿元，年均增长30.6%。社会消费品零售总额由2006年的33.98亿元提高到2011年的83.4亿元，年均增长19.7%。

——三次产业稳步发展。预计三次产业结构比由2006年的20.3：39.8：39.9调整为2011年的15.0：38.1：46.9。农业基础地位不断巩固。农业产业结构调整力度进一步加大，蔬菜、茶叶、中药材、水果、烤烟等特色经济作物种植面积不断扩大，效益不断提高，畜牧业总产值占农林牧渔业总产值的比重提高5个百分点，达到46.1%。农业产业化经营迈出新步伐，柳江集团、雨润黄瀑等龙头企业发展势头良好，农民专业合作社发展到292家。农业机械化程度提高，成为全国丘陵山地农业机械化示范区。新阶段扶贫开发取得阶段性成果，完成易地扶贫搬迁1.83万人，减少农村贫困人口18.78万人。新农村建设取得明显成效，完成134个试点示范村建设。

工业经济规模明显扩大。累计完成工业投资122亿元，纳入地方统计年主营业务收入500万元以上的工业企业从161户增加到226户，预计2011年完成工业增加值72.3亿元，是2006年的3.4倍。以煤炭、电力为主的能源原材料产业发展迅速，原煤产量逐年增加，电力保障能力增强，水泥产量实现翻番；以航空、汽车为重点的装备制造业稳步发展，民用航空产业国家高技术产业基地、青年莲花年产30万辆轿车生产基地落户安顺；以食品、药品为重点的特色轻工业发展加快，百灵集团成功上市，安酒、平坝酒等白酒产业焕发生机。产业园区建设全面提速，安顺经济技术开发区、西秀工业园区、夏云工业园区、普定循环经济工业基地等园区建设力度加大，入驻企业216户，产业聚集效应初步显现。

以旅游业为龙头的第三产业发展迅速。黄果树国家公园启动建设，“半边街”搬迁基本完成。龙宫、格凸河、屯堡文化旅游区、花江大峡谷、关岭古生物化石群国家地质公园等景区深度开发加快。黄果树瀑布节成为国内极具影响力的旅游节庆活动。坝陵酒店、万绿城假日酒店等一批高星级酒店投入使用，商务酒店发展迅速，综合服务接待能力进一步提高。预计2011年，全市接待游客1722.5万人次，实现旅游收入180亿元，分别是2006年的2.33倍和4.86倍。在旅游业的带动下，金融保险、交通物流、邮电通信、信息服务、酒店餐饮、房地产等第三产业快速发展。

——基础设施日益完善。以高速公路为重点的交通基础设施建设进一步加强。全市高速公路通车里程136公里，在建里程160公里。沪昆高速公路安顺段建成通车，普定至安顺及安顺西绕城高速公路年底全线通车，惠水至兴仁、六枝至镇宁高速公路建设稳步推进，安顺至紫云高速公路将于年前开工建设。建成通乡油路530公里、通村公路3422公里，实现所有乡镇通油路（水泥路），所有行政村通公路。黄织铁路建成通车，长昆快速铁路建设进展顺利。500吨级泊位的坝草码头改扩建工程竣工。黄果树机场达到国家4C级标准，新开通安顺至广州航线。以解决工程性缺水为重点的水利设施建设取得新成绩。黔中水利枢纽、安西灌区、“引千入虹”等重点水利工程建设顺利推进，鲁嘎水库、油菜河水库工程年底竣工，治理病险水库77座，建成烟水配套工程173个，新增有效灌溉面积42.95万亩，解决了85.08万人的饮水安全问题。电力、通信等基础设施建设不断加强，实现农村户户通电，20户以上的自然村通广播电视，所有行政村通移动电话。

——城乡面貌明显改变。完成了《安顺市城市总体规划（2009-2030年）》、《安顺市土地利用总体规划（2006-2020年）》修编工作，中心城市详规覆盖率达80%以上，县城详规覆盖率达60%以上。中心城市建设步伐加快，新区开发和旧城改造同步推进，建成区面积由2006年的26.7平方公里扩展到2011年的33平方公里。中华东路、东二环路、龙泉路、龙青路、体育路建成通车，中华西路、武当路、北山路等城市主次干道建设进展顺利，完成一批城区主干道白改黑工程和小街小巷治理工程。城市供水管网改造、电网入地、燃气普及工作稳步推进，金钟、若飞、南马、国际佳缘等一批城市广场建成开放。安顺至普定、平坝马场至花溪党武城市快速干道启动建设。积极推动贵安一体化发展，启动贵安新区建设。各县城加快发展，城市功能不断完善，辐射带动能力明显增强。小城镇规划建设稳步推进，一批富有地方特色、具有一定产业基础的小城镇呈现出蓬勃发展的态势。完成高速公路沿线房屋整治5554栋、村庄整治51个，高速公路沿线面貌焕然一新。生态环境明显改善，全市森林覆盖率达到39%，综合治理石漠化面积300平方公里，治理水土流失面积513平方公里。市和各县城污水处理厂建成并投入运行，各县城垃圾填埋场建设扎实推进。完成主要污染物减排目标。“三创”、“整脏治乱”等活动深入开展，成功创建省级卫生城市和全省文明城市创建工作先进城市。

——改革开放全面推进。深化安顺多种经济成分共生繁荣试验区改革，鼓励、支持和引导非公有制经济发展，预计2011年非公有制经济增加值占全市生产总值的比重达53.9%。市县乡三级政府机构改革顺利完成，事业单位改革稳步推进，市属国有企业改制基本完成，农村综合配套改革和集体林权制度改革深入推进。努力提高投融资能力，组建了城投、交投、工投、农林水投等投融资公司。文化体制改革走在全省前列，文化市场综合执法改革、媒体改革、国有文艺院团转企改制顺利完成。启动医药卫生体制改革，基本药物制度得到全面落实。开展多种形式的对外交流，与珠三角、长三角、京津唐、成渝等地区，以及对口帮扶城市和友好城市的交流与合作进一步加强，“九顺合作”、“农安合作”、“民安合作”、“致安合作”取得明显成效。招商引资工作力度不断加大，五年共引进项目1278个，实际到位资金367亿元。

——人民生活持续改善。全面贯彻落实促进就业再就业的各项政策措施，累计新增城镇就业9.7万人，城镇登记失业率控制在4.5%以内。覆盖城乡居民的社会保障体系框架初步形成,社会保险覆盖面逐步扩大，新型农村养老保险、城镇居民养老保险和被征地农民养老保险试点工作启动实施，城镇居民医疗保险参保率达到90.7%，新型农村合作医疗参合率达到97.5%。城乡低保实现应保尽保，保障标准逐年提高。保障性安居工程建设成效显著，建成廉租住房24万平方米，经济适用房47万平方米，完成农村危房改造63355户。城乡居民收入不断增加，预计城镇居民人均可支配收入由2006年的8590元增加到2011年的15954元，年均增长13.2%；农民人均纯收入由1984元增加到3949元，年均增长14.8%。

——社会发展和谐共进。各级各类教育全面发展，“两基”顺利通过国家验收并得到巩固提高，市二中新校区建成招生，安顺职中被认定为国家级重点中职学校，安顺职院通过教育部高等职业院校人才培养工作评估，安顺学院特色办学迈出新步伐。科技事业取得新进展，安顺市被列为全国科技进步先进市和全国知识产权试点城市。文化事业和文化产业发展呈现良好势头，创作了《唐二闹春》、《我的巴 我的家》等一批具有地方特色的优秀文艺作品。全民健身运动广泛开展，黄果树半程马拉松、紫云格凸河攀岩挑战赛成为具有较大影响力的国际赛事。覆盖市县乡村四级的医疗卫生服务体系基本建立，疾病预防控制体系进一步完善，人民群众“看病难、看病贵”的问题得到缓解。统筹抓好人口和计划生育工作，全市人口自然增长率从2006年的7.1％。下降至2011年的6.3％。。加强和创新社会管理，强化社会矛盾纠纷排查疏导，畅通信访渠道，及时化解不稳定因素。安全生产监管体系不断完善，安全生产形势总体稳定。深入开展“平安安顺”创建工作，城市监控网络体系逐步完善，学校安全保卫工作不断加强，社会大局和谐稳定。高度重视隐蔽战线工作。全面落实民族宗教政策。不断加强国防建设，成功创建省级双拥模范城。老干、档案、史志、外事、侨务、对台、人防、气象等工作进一步加强。

——行政效率大幅提升。自觉接受人大的法律监督和工作监督，自觉接受政协的民主监督。五年来，累计办理人大代表建议456件，政协委员提案854件，做到了件件有答复，事事有回音。认真听取各民主党派、工商联和无党派人士提出的意见和建议，积极支持工会、共青团、妇联、科协、残联、红十字会等人民团体依法按章履行职责。全面贯彻《政府信息公开条例》，加快电子政务建设，切实推进行政权力公开、透明、阳光运行。大力精简行政许可事项，市级行政许可事项由522项减少到101项，办理时限从19.24个工作日压缩到7.5个工作日，将所有审批事项纳入市政务服务中心办理，实行“一站式”办公，“一条龙”服务。应急管理工作进一步加强，预防处置突发公共事件的能力得到提高。严格落实《廉政准则》和党风廉政建设责任制，加大反腐倡廉力度，加强对重点环节和重点部位权力行使的制约和监督，切实做到廉洁、勤政、务实、高效。

2011年，全市经济社会呈现出加快发展的良好势头，是近年来发展得最好最快的一年。预计全市生产总值增长16%；财政总收入和地方财政一般预算收入分别增长25.1%和25.5%；全社会固定资产投资增长132%；年主营业务收入500万元以上工业企业增加值增长23%；城镇化率提高1.5个百分点；招商引资实际到位资金增长205%；社会消费品零售总额增长19%；农民人均纯收入实际增长12%，城镇居民人均可支配收入实际增长10%；单位生产总值能耗下降3.23%；“十大民生工程”顺利完成。创先争优、“三个建设年”、“四帮四促”等活动成效显著。

各位代表，过去五年取得的成就来之不易，这是省委、省政府正确领导和省直各部门大力支持的结果，是全市各族干部群众抢抓机遇、艰苦奋斗的结果。在此，我代表市人民政府，向为安顺经济社会发展付出辛勤劳动和作出积极贡献的全市广大工人、农民、知识分子、干部，向各民主党派、工商联、无党派人士和各人民团体，向驻安人民解放军、武警官兵、公安政法干警、民兵和预备役人员，向所有关心和支持我市改革开放和现代化建设的各界人士，表示衷心的感谢并致以崇高的敬意！

在充分肯定成绩的同时，我们也要清醒地看到，当前我市经济社会发展离省委、省政府的要求和人民群众的期盼，还有不小差距，还面临不少困难和问题。主要是：发展速度相对较慢，经济总量较小，产业结构单一、聚集能力弱；城市建设相对滞后，管理水平不高，辐射带动能力不强，统筹城乡发展压力较大；基层基础相对薄弱，征地拆迁、项目建设等引起的矛盾纠纷依然突出，民生工作还存在许多薄弱环节，人民群众安全感和满意度不高；发展环境不优，行政效率较低，一些长期影响和制约我市发展的体制机制性问题尚未得到根本解决；执行力和落实力还需进一步提高。这些困难和问题，我们将采取更加扎实有效的措施，认真加以解决。

二、今后五年目标任务

今后五年，是我市建设全面小康社会的关键时期，也是我市实现增比进位、赶超跨越的历史攻坚期，更是我市深入实施新一轮西部大开发战略、建设黔中经济区的重要机遇期。国务院即将出台促进贵州加快发展的意见，把贵阳、安顺作为黔中经济区的核心城市，安顺的发展将获得国家层面的有力支持；省委、省政府即将出台支持安顺经济社会加快发展的意见，为我们加快发展提供了政策、资金和项目的有力支撑；安顺广大干部群众谋发展、盼发展的强烈愿望，为我市加快发展提供了强劲的内生动力。只要我们紧紧围绕“加速发展、加快转型、推动跨越”的主基调，按照“抓机遇、提速度、上台阶”的总要求，坚定信心，明确目标，干字当头，敢字为先，拼字求胜，未来五年就会成为我市改革开放以来发展最好最快的时期，成为城乡人民生活改善最为显著的时期，我们将在新的起点上谱写出更加壮丽美好的篇章！

按照市第三次党代会的部署，未来五年我市经济社会发展的奋斗目标是：全市生产总值年均增长28%以上（未扣除物价因素），确保2016年达到980亿元以上；全社会固定资产投资年均增长55%以上，累计完成5700亿元以上；地方财政一般预算收入年均增长30%以上，2016年达到92亿元以上；招商引资到位资金年均增长50%以上，累计完成2900亿元以上；农民人均纯收入年均增长20%以上，城镇居民人均可支配收入年均增长18%以上，2016年分别达到9830元和36500元以上。力争固定资产投资和招商引资增速保持在全省前列，主要经济指标在全省的排名上升一到两位，综合实力上一个新台阶，城乡面貌上一个新台阶，人民生活水平上一个新台阶。

（一）全力以赴扩大固定资产投资规模，突出抓好项目建设，促进经济发展大跨越

把项目建设作为促进经济跨越发展的重要抓手，力争五年实施项目3000个以上，项目总投资达到8000亿元以上。

加强项目前期工作。科学筹划和编制好一批产业链长、产业幅宽、带动性强的大项目、好项目，确保项目库投资规模保持在10000亿元以上。加大项目前期工作经费投入力度，确保各级财政用于项目前期工作的经费逐年递增。完善和落实项目前期工作责任制，抓好项目前期规划、设计、申报及储备工作，提高项目的成熟度和可行性，形成投产一批、建设一批、储备一批和谋划一批的项目接续机制。

加大土地和资金保障力度。统筹用地指标，积极争取增量，加强土地开发复垦，清理闲置土地，用好土地增减挂钩政策，积极申报“以质抵量”试点，全力保障项目建设用地。增强投资强度，提高节约集约用地水平。创新投融资理念，发挥政府投资的引导作用，用好金融机构贷款授信额度，大力开展项目融资，力争金融机构贷款余额年均增长30%以上。充分发挥投融资平台的作用，积极做好债券发行、基金发行和股权融资等工作。采取BOT、TOT、BT等方式拓宽融资渠道，引导社会资金广泛参与项目建设。

加快项目建设步伐。重点抓好重大交通、水利等基础设施项目的实施。全力配合做好境内长昆快速铁路、黄百铁路等铁路建设。新建高速公路263公里以上，实现县际道路达到二级以上技术标准，70%以上的行政村通油路或水泥路，所有自然村通公路。实施贵阳至安顺城际轨道交通项目。扩建黄果树机场，开通更多航线。开辟南下出海通道，组建坝草码头航运公司。继续配合做好黔中水利枢纽工程建设，加强农田水利基本建设，继续实施烟水配套工程，推进“五小水利”工程建设，加快灌区续建配套和改造，建成骨干水源工程16个，治理病险水库76座，新增有效灌溉面积36万亩，解决农村饮水安全42万人以上，基本解决工程性缺水问题。建设“数字安顺”，稳步推进“三网融合”，实现农村宽带到村、城市光纤到户、通信信号全覆盖。

（二）全力以赴实施工业强市战略，突出抓好园区发展，促进工业经济大提速

实施工业发展“三百工程”，力争到2016年，工业总产值突破1660亿元，工业增加值占全市生产总值的比重达40%以上。

建成百亿级园区。加快推进园区基础设施建设，确保省级以上产业园区达到“七通一平”，市级产业园区达到“五通一平”。大力推进标准化厂房建设和公共服务平台建设，提升园区公共配套能力。创新园区投融资体制，积极与发达地区、大企业、大集团等合作，共同建设园区，实现利益共享。引导企业入驻园区，培育一批规模大、带动性强的企业集团，形成产业集聚。积极申报一批省级开发区，力争安顺经济技术开发区申报成为国家级开发区。到2016年，民用航空产业国家高技术产业基地产值达到500亿元以上，西秀工业园区、黎阳高新技术产业园区、黔中新兴产业园区产值分别达到300亿元以上，普定循环经济工业基地产值达到200亿元以上。

打造百亿级产业。大力发展飞机制造、客机改货机、航空维修、航空培训等民用航空产业，建成全国通用飞机、无人机生产和试训基地；大力发展汽车整车及零部件制造，建成年产30万辆轿车生产基地一期工程；大力发展专用设备和特种机械制造，形成高端装备制造体系。充分发挥我市能源和矿产资源组合优势，积极申报工业直供电试点，推进煤—电—冶（化）联产联营，优化铝及铝加工、铁合金等产业，重点发展煤化工、无机化工、环保建材等，建成全国精细碳酸钡生产和研发基地，努力延长产业链，实现资源的经济效益本地最大化。大力发展以苗药为核心的制药业，振兴安顺白酒，加快发展肉制品、调味品和精制茶等地方名优产品，积极推进蜡染、脸谱、屯堡银器、仿真古生物化石等特色旅游商品开发与生产。鼓励发展信息技术、生物技术、新材料、新能源等高新技术产业。到2016年，装备制造业产值达到600亿元以上，原材料产业、高新技术产业产值分别达到300亿元以上，能源产业、特色轻工业产值分别达到200亿元以上。

培育百亿级企业。以资源优化配置为基础，以资产为纽带，培育发展一批成长性好、带动力强、关联度高、具有较强市场竞争力的骨干企业。积极引进战略投资者，吸引跨国公司、中央企业、国有大型企业、有实力的民营企业参与全市工业经济结构布局调整和企业组织结构调整，促进工业产业结构优化升级，发展壮大一批大企业，鼓励企业集团化发展，支持有条件的企业上市融资。到2016年，培育亿元级企业50家、十亿元级企业10家、五十亿元级企业5家、百亿元级企业3家。

（三）全力以赴实施城镇化带动战略，突出抓好中心城市建设，促进城乡面貌大改观

加快推进城镇化进程，确保城镇化率每年提高1.5个百分点以上，到2016年，全市城镇化率达到44%以上。

高起点编制规划。科学谋划和布局城市整体功能，提高城市规划水平。加快推进城市各类详规、专项规划编制工作，完善县、乡、村各级规划，实现规划全覆盖。高标准抓好城市重要区域和重要节点景观规划设计。加强规划的统筹协调，有序实施各项规划，避免重复建设。加大规划执法力度，严厉打击各类违法建设，全力保证规划的权威性和严肃性。

高品位打造中心城市。加快推进新区开发，完成新火车站、市行政中心等建设，带动新火车站片区、北部新区、西秀新区快速发展。完善中心城市路网结构，建成城市道路89公里，迅速拉开城市框架，扩大城市规模，使中心城市面积达到62平方公里，人口规模达到60万人以上。大力推进旧城改造，基本完成城中村、棚户区及城市重要节点改造工作。全面完成中心城市电网入地工程，改造城区路灯、景观灯，打造亮丽城市景观。大力实施城市绿化工程，推广立体绿化，打造绿色通道和生态景观带，建成区绿化覆盖率达到36%以上。规划建设独具特色的黄果树大街城市景观大道、特色商业街区、休闲娱乐街区、餐饮美食街区、历史文化街区，建成一批开放式公园及大型城市文化广场，提升山水园林城市形象，完善旅游城市要素，丰富历史文化城市内涵，使城市面貌焕然一新。

高速度建设县城和小城镇。加快城市快速干道建设，实现中心城市与平坝、普定、镇宁一体化发展，拓展城市发展空间。推动普定进入全省经济强县行列。推进关岭顶云新区及紫云新城建设，把关岭、紫云建设成为区域性中心城市。完成黄果树新城建设，把黄果树建设成为区域旅游集散中心。推进贵安一体化发展，加快贵安新区规划建设，打造内陆开放型经济示范区。着力培育一批基础设施条件好、生态环境优良、具有一定产业基础的旅游、工贸、商业服务型特色小城镇。积极申报设立虹阳区，推进平坝、普定撤县建区工作，加快“镇改办”、“村改居”步伐。

高标准完善城镇功能。完成中心城市供水二期工程，完善各县城、乡镇供水保障体系，城镇供水普及率达到100%。加强集中式饮用水源地保护，确保水质全面达标。完善城镇公共交通运输体系，规划建设一批停车场、人行天桥和地下人行通道，提高交通组织能力，缓解城市交通压力。完善城市供气管网，确保城市燃气化率达到70%以上。完善中心城市和县城污水收集、处理、再生利用系统，确保县城以上污水处理率达到80%以上。大力实施能源改造和清洁能源建设工程，推进大气污染联防联控，确保空气质量优良率保持100%。加快垃圾收运系统建设，推行垃圾分类处理，确保县城以上生活垃圾无害化处理率达到85%以上。

高效能管理城市。按照统一领导、分级管理、重心下移、区街为主的总体要求，建立健全城市管理体制机制，推行数字化管理，提高城市管理工作效率和精细化水平。深入开展“三创”和“整脏治乱”工作，到2016年，成功申报国家卫生城市，成功申报国家文明城市创建工作先进城市，启动国家环保模范城市申报工作。

（四）全力以赴推进改革开放，突出抓好招商引资，促进发展活力大增强

深化安顺多种经济成分共生繁荣试验区改革，全面扩大对内对外开放，不断激发创新活力，努力建设开放安顺。

继续深化各项改革。抓好行政管理体制、投融资体制、财税体制、医药卫生体制等重点领域和关键环节的改革。深化人事制度改革，推进事业单位分类改革，加快形成合理有序的工资收入分配格局。深化农村综合配套改革，稳步推进农村土地有序流转，完善农村发展的体制机制。深化户籍制度改革，探索建立一元化城乡户籍管理模式。全面推动社会领域的改革，提高基本公共服务均等化和社会管理科学化水平。完善国资营运监管体系，提高国有资产营运效益。组建安顺市农村商业银行，发展一批村镇银行和小额贷款公司，引进2家以上股份制商业银行，建成股权投资和产权交易市场。

全面扩大对内对外开放。夯实招商引资项目库,实行动态管理，确保招商引资项目库规模保持在5000亿元以上。进一步完善招商引资优惠政策，严格执行招商引资目标考核制度，创新招商引资方式，以商招商，全民招商，构建“大招商”格局。优化投资环境，构建“务实、创新、高效、廉洁”的服务体系，营造“亲商、安商、扶商、助商”的良好氛围。积极承接产业转移，争取体量大、带动性强、技术水平高、发展后劲足的大企业大项目落户安顺。积极申报建设综合保税区、国家级出口基地和无水港，争取设立海关及出入境检验检疫机构。重点培育一批出口加工企业，鼓励优势产品扩大出口。积极“走出去”，深化与经济发达地区、港澳台及东南亚地区的经贸文化交流，积极参加广交会等全国性的大型会展活动，继续加强与黔中经济区其他城市、对口帮扶城市、友好城市的合作。

大力发展非公有制经济。认真落实促进非公有制经济发展的政策措施，激发全民创业热情，动员各类市场主体参与安顺经济建设。鼓励、支持和引导民间资本、非公有制企业进入能源、基础设施、政策性民生工程和社会公用事业等行业。采取资本金补助、担保贷款、税收返还等政策，鼓励扶持小微企业发展。建立非公有制优质企业库，实施中小企业成长工程，帮助有发展潜力的中小企业做大做强。力争到2016年，非公有制经济增加值占全市生产总值的比重达到65%以上。

建设创新型城市。完善科技创新体制机制，着力培育高水平的研发机构和队伍，着力推动企业技术创新、产业技术创新和社会发展创新，着力提升科技创新和成果转化能力，实现创新成果专利化、资本化、市场化，大幅提升科技进步对经济增长的贡献率。大力实施黔中英才培养等13个人才发展重点工程，完善人才引进、培养、使用和管理的体制机制，加快培养和集聚高层次科技人才、高素质管理人才、高技能实用人才。坚持尊重知识、尊重人才，为各类人才创新创业营造良好环境，让一切劳动、知识、技术、管理和资本的活力在黔中大地竞相迸发！

（五）全力以赴发展旅游产业，突出抓好转型升级，促进第三产业大提升

按照国际公认的“8A”标准，努力打造国际旅游目的地城市，到2016年，旅游总收入达到680亿元，第三产业增加值达到350亿元。

打造国际标准精品景区。加快黄果树国家公园建设步伐，以黄果树大瀑布为核心，以龙宫、格凸河、屯堡、花江大峡谷为重点，建设平坝至关岭黄金旅游走廊。整合天龙、七眼桥、旧州、九龙山等旅游资源，形成大屯堡旅游区。争取黄果树成功申报世界自然遗产。申报一批国家级风景名胜区和4A、5A级旅游景区。加强旅游宣传促销，积极拓展国内、国际市场。把黄果树瀑布节打造成国际知名的旅游节庆活动。积极培育和引进一批大中型旅行社，推出一批旅游精品线路，吸引更多国内外游客到安顺旅游。到2016年，接待游客达到6400万人次以上，游客平均停留时间提高到3天以上。

加快旅游发展方式转变。建成10家以上五星级酒店和一批四星级酒店，加快发展商务酒店和休闲度假设施。大力建设高端休闲度假、极限运动、民族风情等旅游区，打造集观光、科考、探险、文化、红色旅游、乡村旅游为一体的多元化旅游产品发展格局，实现从观光游向休闲度假游质的飞跃。加快旅游公路、停车场和游客服务中心等基础设施建设，建成黄果树、龙宫、格凸河旅游环线公路和屯堡景区、关岭古生物化石群国家地质公园旅游专线公路。加快建设数字化、多语种的旅游咨询服务体系，推进“智慧旅游”，建设数字旅游示范区。

加快发展现代服务业。重点建设黄桶—幺铺等物流园区，扶持优强物流企业加快发展，把安顺建成重要的物流节点城市。大力发展金融保险、交通运输、信息服务等生产性服务业，不断提升商业购物、社区服务、社会养老、房地产等生活性服务业，支持发展法律、会计、审计、培训等中介服务机构，培育优质服务品牌。

（六）全力以赴加快农业现代化进程，突出抓好结构调整，促进农村经济大发展

始终把“三农”工作作为全部工作的重中之重，全面落实各项支农惠农政策，促进农村发展、农业增效、农民增收。

优化农业产业结构。稳定粮食生产，着力提高单产。大力发展特色种植业，建成100万亩蔬菜基地、50万亩茶叶基地、50万亩中药材基地、30万亩精品水果基地、60万亩核桃基地、20万亩烤烟基地、20万亩安顺金刺梨基地。大力发展现代生态畜牧业，建成300万头生猪养殖基地、500万羽生态蛋鸡基地、3000万羽肉禽生产基地，确保畜牧业总产值占农林牧渔业总产值的比重提高到50%以上。大力扶持农民专业合作社，培育一批大型龙头企业。推进全国丘陵山地农机化示范区建设，大力提升农业机械化综合水平。加快现代农业社会化服务体系、农技推广体系、农产品质量安全体系和农产品流通市场体系建设。积极申报建设国家山区现代农业示范区，支持镇宁、关岭、紫云申报国家农业综合开发县和小型农田水利试点县。

深入推进新农村建设。加快基础设施建设向下延伸，大力实施水、电、路、气、房、环境改善“六到农家”工程，积极创建“四在农家”示范点。鼓励支持城市周边、旅游景点、公路沿线发展休闲观光农业，建设农业经济示范带。认真实施民族事业发展十大推进计划，打造一批有特色的民族村寨。把村庄整治从公路沿线向纵深推进，到2016年完成80%的村庄整治工作。推进农村环境综合整治，改善农村生产生活条件，切实解决农村垃圾乱倒、粪土乱堆、污水乱排、柴草乱垛、畜禽乱跑的“五乱”现象，实现道路硬化、村庄绿化、重要节点亮化。

扎实推进生态建设。围绕水利建设、生态建设、石漠化综合治理“三位一体”规划，大力实施退耕还林、荒山造林、天然林保护、珠防等重点林业工程，确保森林覆盖率每年增加1个百分点以上，到2016年全市森林覆盖率达到45%，完成石漠化综合治理700平方公里，治理水土流失面积580平方公里。

（七）全力以赴提升文化软实力，突出抓好文化产业，促进黔中文化大发展大繁荣

深化文化体制改革，不断解放和发展文化生产力，到“十二五”期末，文化产业增加值占全市生产总值的比重达到6%以上。

大力发展文化产业。推进“一核、七园、八大文化旅游产业组团”建设，加快建设黔中国际屯堡文化生态园、蜡染艺术园、穿洞文化园等一批重点文化产业园。着力构建特色鲜明的黔中文化品牌体系，努力打造夜郎、牂牁、三国、屯堡、红色、三线等文化品牌，争取屯堡文化成功申报为世界非物质文化遗产。创作《关山度若飞》、《亚鲁王》等一批重点题材文艺精品，加强安顺地戏、铜鼓十二调等国家级非物质文化遗产保护和开发。加大文化企业培育力度，扶持一批文化骨干企业发展壮大。

加快发展公益性文化事业。构建城乡一体化公共文化服务网络。新建安顺大剧院、文化馆、图书馆、博物馆、档案馆、体育馆（场、中心）、青少年活动中心、老年活动中心、会展中心等一批文化体育设施。实现县县都有文化馆、图书馆和体育场馆，实现乡镇综合文化站、文化小广场和公共电子阅览室全覆盖，20户以下自然村户户通广播电视。完善覆盖城乡的文化信息资源共享网络。逐步实现公共文化设施免费开放，全面提高公共文化服务水平。

加强社会主义核心价值体系建设。大力弘扬以爱国主义为核心的民族精神和以改革创新为核心的时代精神，树立和践行社会主义荣辱观，加强社会公德、职业道德、家庭美德、个人品德教育，加强和改进未成年人思想道德建设，在全市上下倡导“爱国、敬业、诚信、友爱”的价值取向。深入开展形式多样的精神文明创建活动，不断提高市民文明素质和社会文明水平。

（八）全力以赴加强社会公共服务，突出抓好民生工程，促进人民生活水平大提高

民生大于天，民生无小事。坚持把民生摆在更加突出的位置，实现发展成果由人民共享，让全市人民过上更加幸福、更有尊严的生活！

切实保障和改善民生。把就业作为最根本的民生，实施积极的就业政策，加大培训力度，提高劳动者素质，多渠道开发就业岗位，确保每年新增就业人数2.2万人以上，城镇登记失业率控制在4.5%以内。把社保作为最普遍的民生，加快推进覆盖城乡的社会保障体系建设，提高城镇基本养老、基本医疗保险参保率和新型农村合作医疗参合率，进一步完善城乡居民最低生活保障制度，逐步提高保障水平。把教育作为最长远的民生，实施好《安顺市中长期教育改革和发展规划纲要（2010-2020年）》，确保到2016年，九年义务教育巩固率、高中阶段毛入学率、高等教育毛入学率和学前三年毛入学率，分别达到85%、63%、27%和60%以上。把住房作为最急迫的民生，进一步加快保障性住房建设，确保到2016年，新建4万套以上保障性住房，全面完成农村危房改造。把扶贫作为最突出的民生，抢抓国家启动实施《中国农村扶贫开发纲要（2011-2020年）》的战略机遇，大力推进开发式扶贫和产业化扶贫，确保到2016年，全市贫困人口减少到46万人以下（按新标准测算）。

大力发展各项社会事业。提高医疗卫生服务能力，加快综合医院建设，推进县乡村三级卫生服务网络和城市社区卫生服务体系建设，努力为群众提供安全、有效、方便、价廉的医疗卫生服务。加强人口和计划生育工作，全市人口出生率、人口自然增长率分别控制在12.5％。和6％。以内。积极应对人口老龄化问题，加快完善社会养老体系。加大殡葬改革力度，提高管理服务水平。广泛开展全民健身活动，提高人民群众健康素质。巩固和发展军政军民团结，促进国防建设与经济建设协调发展。做好民族宗教、老干、档案、史志、外事、侨务、对台、人防、气象等方面的工作。

（九）全力以赴加强和创新社会管理，突出抓好“平安安顺”建设，促进社会和谐进步

着力提高社会管理科学化水平，有效保障人民群众合法权益，形成民主法治、公平正义、诚信友爱、充满活力、安定有序、人与自然和谐相处的社会格局。

完善社会管理体系。加快形成和完善党委总揽全局，政府负责实施，社会各方协作，公众广泛参与的社会管理格局。加强社区建设，健全基层群众自治机制，完善管理和服务体系。引导和强调企业承担社会责任，发挥各类商会、学会、行业协会等社会组织在社会管理中的重要作用，充分调动社会各方面力量共同参与社会管理。深入推进“六五”普法工作，营造良好法治环境。

推进“平安安顺”建设。切实维护群众权益，加强信访工作，畅通和规范群众诉求表达、利益协调、权益保障渠道。创新社会矛盾化解机制，建立争议排查调处制度，完善大调解工作格局。实行社会稳定风险评估制度，从源头上预防和减少社会矛盾。建立健全自然灾害、事故灾难和突发公共事件的预防预警和应急管理体系，提高防灾减灾和危机管理能力。加强食品药品安全监管。健全安全生产监管体系，落实责任制，确保安全生产形势稳定。完善社会治安防控体系，加强城乡社区警务、群防群治等基层基础建设，强化社会治安综合治理，全力维护社会和谐稳定。

三、2012年经济社会发展的主要目标和任务

2012年是新一届政府履行职责的开局之年，我们将迎来党的十八大和省第十一次党代会的召开，做好明年的各项工作，意义十分重大。2012年全市经济社会发展的主要预期目标是：生产总值增长26%以上（未扣除物价因素）；全社会固定资产投资增长55%以上；地方财政一般预算收入增长30%以上；社会消费品零售总额增长20%；城镇居民人均可支配收入增长18%，农民人均纯收入增长20%；城镇登记失业率控制在4.5%以内；人口自然增长率控制在6.2％。以内；居民消费价格总水平涨幅控制在全省平均水平；完成省下达的节能减排任务。

围绕上述目标，着力抓好以下十个方面的工作：

（一）着力抓好项目建设，在扩大固定资产投资规模上取得新突破

加快项目编制工作，扩充项目库，确保全市项目库投资规模达到10000亿元。市级安排5000万元项目前期工作经费，推动项目顺利落地。扎实抓好交通基础设施建设，配合做好长昆快速铁路、黄百铁路的建设工作，全面推进长昆快速铁路我市境内三个站点建设；配合做好惠水至兴仁高速公路建设，确保惠水至紫云段建成通车，实现县县通高速；配合做好六枝至镇宁高速公路建设工作，扎实推进安顺至紫云高速公路建设，开工建设普定至织金高速公路；建成安顺至普定城市快速干道，推进平坝马场至花溪党武城市快速干道建设，开工建设黔中新兴产业园区马场桐木寨至狗场城市快速干道；开展贵黄高等级公路改扩建城市快速干道、大西桥中所经白云肖家至花溪城市快速干道和贵阳至安顺城际轨道交通项目前期工作；加快县乡公路建设，建成600公里通村油路（水泥路）；加大航线培育力度，力争开通安顺至北京、上海等地航班；启动坝草码头出海航运。加快水利基础设施建设，继续做好黔中水利枢纽、安西灌区渠道和“引千入虹”工程建设，开工建设石朱桥水库和新场河水库，力争开工建设黄家湾水利枢纽工程和坝陵河水库，治理病险水库24座，加快农田水利工程、农村饮水安全工程和烟水配套工程建设，新增有效灌溉面积8万亩，解决12万人饮水安全问题。通过项目建设的强力推进，确保2012年固定资产投资超过400亿元，增速排在全省前列。

（二）着力抓好规划建设管理，在加速城镇化进程上取得新突破

高标准、高起点完成中心城市景观风貌规划、北部新区详规等规划，所有县城详规覆盖率达到100%，全面完成乡镇、村寨规划编制工作。强化规划的统一性和权威性，严查严处违法建设行为，坚决遏制违法建设泛滥的势头。全力推进中心城市“一轴线四片区”开发建设，大力实施黄果树大街整治工程，在沪昆高速公路安顺东出口片区建成城市园林精品的道路绿化景观，在南出口片区打造极具地方特色的风情园，把黄果树大街打造成城市景观大道。推进二环路建设，建成武当路、北山路、安普路等一批城市主次干道，全面完成城区主干道白改黑工程。实施中华西路等棚户区改造和管元村等“城中村”改造项目，建成若飞公园、虹山湖城市公园，推进历史文化街区建设。加快北部新区、新火车站片区和西秀新区路网等基础设施建设，启动建设新行政中心。开工建设市级交通枢纽组织管理中心、东客运站、北客运站，加快市区人行天桥、地下人行通道和公共停车场建设，缓解城区交通压力。启动中心城市供水二期工程。支持平坝迎宾大道二期、镇宁沿河路、关岭县城至顶云新城快速干道、惠水至兴仁高速公路紫云联络线等项目建设。着力提高城市管理水平，加强城市管理执法队伍建设，树立城市管理执法新形象。加大市政设施巡查修复力度，保障居民生活便利。多渠道筹集城镇建设资金，确保城镇建设投资增幅超过60%，城镇化率达到38%以上。

（三）着力抓好产业园区建设，在加快工业发展上取得新突破

加快园区规划建设，明确各产业园区功能，全面完成园区发展规划和建设规划编制工作。市级筹集10亿元资金，用于加快园区基础设施建设。民用航空产业国家高技术产业基地、夏云工业园、西秀工业园达到“五通一平”，其他园区逐步达到“三通一平”，力争西秀工业园、夏云工业园、普定循环经济工业基地、镇宁特色轻工产业园区、黔中新兴产业园区成功申报省级重点产业园区（开发区）。推进黔中新兴产业园区与江苏省合作共建国家级信息产业园区。鼓励和引导企业向园区集中，实现集群发展，确保园区新增规模以上企业30家，规模以上企业总产值达到150亿元。全力推进工业项目实施，全面完成煤炭生产企业兼并重组工作，组建一批年生产能力400万吨的大型煤炭企业集团，开工建设安顺电厂三期和盘江大型带式输送机输煤工程，推进青年莲花轿车生产基地一期工程和百灵阿莫特飞机项目建设，认真抓好黄果树铝业二期技改、红星钡盐技改、永昌水泥、华兴玻璃和黄果树金叶烟叶薄片等一批重点项目建设，确保全年完成工业投资160亿元，增长129%以上。全力保障煤、电、油、运等生产要素，加强工业经济预测分析和运行调度，千方百计扩大产能，确保2012年工业增加值增长28%以上。

（四）着力抓好招商引资，在扩大开放上取得新突破

继续深化改革开放，大力发展非公有制经济，鼓励以创业带动就业，扶持新增一批小微企业，扎实推进“万户小老板”工程，扶持中小企业扩大生产规模，新增规模以上非公企业30家。切实加大招商引资力度，完善招商引资工作责任制和考核奖励办法，制定市直部门向上争取支持的考核奖励措施，严格兑现奖惩。完善招商引资项目库，确保入库招商引资项目投资规模达到5000亿元以上。加强招商引资项目前期工作，完善招商引资项目信息发布平台，创新招商引资方式，充分发挥驻外招商分局作用，在长三角、珠三角等地开展大型招商引资活动，确保2012年招商引资实际到位资金超过270亿元，增速排在全省前列。

（五）着力推进旅游业转型升级，在带动第三产业加快发展上取得新突破

推进黄果树国家公园建设，实施黄果树“半边街”生态恢复工程，支持黄果树旅游集团公司上市。推进开发区多彩文化旅游项目建设。围绕坝陵河大桥，认真做好“大桥世界”项目前期工作。加快郎宫旅游休闲度假区建设步伐。实施关岭古生物化石群国家地质公园和天龙屯堡文化遗产保护项目。抓好虹山等高星级酒店的建设，支持建设一批富有地方特色和民族特色的乡村旅馆。以“多彩贵州，最美安顺”为宣传促销主题，推出以赏花、避暑、休闲为主的旅游产品，3月份办好油菜花旅游节及蜡染艺术节，5月份办好黄果树瀑布节。力争全年接待游客超过2240万人次，旅游收入超过240亿元。大力发展现代服务业，加快黄桶——幺铺物流园区建设，深入推进“万村千乡市场工程”，抓好汽车、建材、小商品等专业市场建设。大力发展交通运输、房地产、金融、酒店餐饮、文化娱乐、健身养生、信息服务、会展等现代服务业，确保2012年第三产业增加值增长18%以上。

（六）着力加强农业产业结构调整，在推进农业现代化进程上取得新突破

稳定粮食生产，优化产业结构，种植蔬菜80万亩、中药材6万亩、水果5万亩、核桃13万亩，新建茶园6万亩，推广安顺金刺梨5万亩，推进鑫龙薏仁米种植基地建设，完成烤烟收购任务25万担。加快发展现代生态畜牧业，确保畜牧业产值增长20%，达到36亿元，占农林牧渔业总产值的比重提高1个百分点以上。完成华西希望集团50万头商品猪养殖基地、唐人神集团种猪场、柳江集团150万羽生态蛋鸡养殖基地等项目建设。扶持发展30个以上农民专业合作社，提高农业生产经营的组织化程度。引进和扶持一批大中型农产品加工企业，推动雨润黄瀑120万头生猪冷鲜肉生产线尽快达产，支持牛来香、百花串等龙头企业扩大产能。规划建设安顺市黔中农产品交易大市场，推进中所国家级定点生猪市场改扩建工程建设。落实好农业部部属单位支持安顺农机化发展框架协议。推进农业生产实现特色化、规模化、商品化、产业化，确保2012年农民人均纯收入超过4740元。

（七）着力深化文化体制改革，在加快文化产业发展上取得新突破

探索建立文化事业单位法人治理结构，引入社会力量参与文化事业单位的发展与内部运营管理。推进转企改制文化企业按照现代企业制度建立健全法人治理结构，培育合格的文化市场主体。全面落实文化改革发展税收、土地、投入等各项优惠政策。完成屯堡文化和生态文化产业集聚区、古生物化石文化产业集聚区等4个文化产业集聚区规划编制工作。加快实施“六个一批”文化工程，力争启动大明屯堡影视文化城项目建设，完成黔中报业中心项目主体工程。建设一批乡镇公共电子阅览室和农民文化家园，实现农家书屋全覆盖，实施调频应急广播“村村响”和20户以下自然村通广播电视工程。扶持发展花灯戏、屯堡地戏等富有地方特色的演艺剧团，组建广电传媒集团、黔中文化产业投资公司。支持黔中早报提高办报水平，实现全省发行，扶持黄果树艺术团等一批文化市场主体做大做强。

（八）着力加强公共服务设施和生态环境建设，在改善城乡人居环境上取得新突破

开工建设安顺大剧院、博物馆、图书馆、规划展览馆、文化活动中心、青少年活动中心、老年活动中心、会展中心，力争启动建设市体育中心。实施亮丽工程，全面完成中心城市路灯、景观灯改造。实施一批城市绿化工程，使中心城市建成区绿化覆盖率提高到28%。每个县城建成一条城市景观大道、一个市民广场、一个城市公园、一个文化体育中心（或图书馆、陈列馆）、一个公共停车场。每个乡镇建成一条市政道路、一个农贸市场、一个群众文化广场、一个体育活动场所、一个垃圾清运系统、一支城镇管理队伍。大力推进村庄整治，完成43个“百村试点”整治工作，创建180个“四在农家”示范点。继续抓好退耕还林、天然林保护等重点林业工程建设，完成营造林30万亩，确保森林覆盖率达到40%以上。完成石漠化综合治理118平方公里、水土流失综合治理120平方公里。加快城镇污水处理及再生利用设施建设，完善中心城市垃圾收运系统，建成医疗废弃物处置中心并投入运行，全面完成各县城垃圾填埋场建设。深入推进“三创”和“整脏治乱”工作，启动全国卫生城市和新一轮省级文明城市创建工作。通过人居环境的不断改善，让人民群众的满意度得到大幅提升。

（九）着力加强民生工程建设，在提高人民生活水平上取得新突破

实施更加积极的就业政策，新增城镇就业2.2万人以上，大力发展劳务经济，转移农村劳动力就业5万人。实施城镇职工增薪计划，着力提高工资水平，确保城镇职工最低工资标准增长14%以上。提高村（社区）干部报酬，超过全省平均水平。继续完善工伤、医疗、生育保险市级统筹工作，扩大新型农村社会养老保险试点范围。提高城镇职工、城镇居民基本医疗保险参保率，新型农村合作医疗参合率稳定在95%以上。建立失地农民和水库移民基本生活保障制度。进一步完善城乡社会救助体系建设，确保城乡低保标准提高10%以上，安排好困难群众的基本生活。加快“一危三棚”改造和保障性住房建设，完成农村危房改造25000户，实施棚户区改造1495户，建成城镇保障性住房5926套，新开工建设城镇保障性住房1643套。切实发挥集团帮扶优势，整合项目资金，加快实施石漠化集中连片特困地区扶贫开发攻坚工程，完成易地扶贫搬迁2500人，减少贫困人口15万人（按新标准测算）。推进各项社会事业加快发展。巩固提高“两基”成果，实施学前教育三年行动计划、高中阶段突破工程和农村寄宿制学校建设攻坚工程，启动安顺职院新校区建设，支持安顺学院加快发展，推动市委党校迁建工作。加强校车规范化管理和学校安全管理。全面实施农村义务教育阶段学校营养改善计划。加强科技创新，确保全国知识产权试点城市顺利通过验收。积极推进公立医院改革，加快市人民医院异地新建工作。加强人口计生工作，实现“双降”目标。建设一批乡镇体育健身工程、村级农民体育健身工程、全民健身路径工程。通过我们的不懈努力，让人民群众的幸福感得到大幅提升。

（十）着力建设“平安安顺”，在加强和创新社会管理上取得新突破

认真做好新形势下的群众工作，整合资源，建立市县乡村群众工作机构，形成纵向到底、横向到边、四级互联的群众工作网络。用群众工作统揽信访工作，变上访为下访，变被动接访为主动服务，加大矛盾纠纷排查力度，把矛盾化解在基层，化解在萌芽状态。强化城乡社区（村）自治和社会自治，突出城乡社区（村）基层组织在建设文明和谐社区（村）中的主体作用。完善劳动关系协调机制，加大劳动保障监察力度。贯彻落实党的民族宗教政策，在全市范围内开展民族团结创建活动。强化以煤矿为重点的安全生产基层基础建设，狠抓安全生产各项责任制的落实。强化食品药品安全监管。完善突发公共事件应急管理机制，切实加强应急处置能力建设。继续深入开展“平安安顺”创建活动，抓好城市报警和监控系统建设，强化治安防控，提高见警率，加大对各种违法犯罪的打击力度，切实让人民群众的安全感得到大幅提升。

四、切实加强政府自身建设

各位代表，新一届政府肩负着承前启后、继往开来，带领全市各族人民奋力加速发展，实现增比进位、赶超跨越的重任。我们将切实转变作风，提高行政效能，努力做团结和谐、干事创业、清正廉洁、敢于担当的表率，决不辜负全市各族人民的殷切期望！

（一）切实加强作风建设

牢固树立执政为民的理念，坚持深入基层和群众，真诚倾听群众呼声，真情关心群众疾苦，真心为群众干实事，努力实现好、维护好、发展好最广大人民群众的根本利益。大力弘扬雷厉风行、真抓实干的良好作风，敢于尝试，勇于担当，宽容失误。坚持高目标引领，高强度推进，高效率落实，不断提高执行力和落实力。建立健全奖惩机制，使发展压力层层传递、工作动力级级提升，激发各级干部干事创业的激情，以实干求业绩，以实绩赢民心。

（二）全面提高行政效能

着力提升干部队伍素质，切实增强抓经济、抓项目、抓工业、抓城建、抓开放的本领，提高政府谋发展的能力，应对复杂局面的能力，为人民谋取更大福祉的能力。优化行政审批流程，简化行政审批环节，凡是可以下放的权力一律下放，凡是可以取消的收费一律取消，凡是可以精简的审批一律精简。进一步加强政务服务中心建设，实施电子政务外网工程。下大力气精简会议、压缩文件，切实把时间和精力放在抓落实上，真正做到知实情、谋实招、重实干、求实效。

（三）深入推进依法行政

严格按照法定权限和程序行使职权，履行职能，建设法治政府。完善政府工作规则和议事规则，建立健全重大决策调查研究、征求意见、专家论证、听证公示、决策评估以及责任追究等制度，努力提高政府的科学决策水平和公信力。自觉接受人大及其常委会的法律、工作监督和政协的民主监督，认真办理人大代表的建议和政协委员的提案，高度重视民主党派、工商联、无党派人士和各人民团体的意见、建议，主动接受新闻舆论和社会公众监督，不断提高政府信息公开水平，让人民赋予的权力在阳光下运行。

（四）加强反腐倡廉建设

认真落实党风廉政建设责任制和领导干部廉洁从政的各项规定，不断完善教育、制度、监督并重的惩治和预防腐败体系，从源头防治和杜绝腐败现象发生，切实提高反腐倡廉科学化水平。加强行政监察、财政监督和审计监督，强化对工程建设、土地出让、行政审批等重点领域的管理和监督。严肃查处各类违法违纪案件，依法严惩腐败分子，大力营造风清气正的政务环境。

各位代表，新的目标鼓舞人心，新的形势催人奋进。新一届政府将高举“发展、团结、奋斗”的旗帜，紧紧围绕建设实力安顺、宜居安顺、开放安顺、平安安顺的目标，干字当头，努力干出安顺特色，全力干出安顺风格，奋力干出安顺速度，强力干出安顺气魄，为实现与全省全国同步进入全面小康社会而不懈奋斗！
